# Transcanal Endoscopic Tympanoplasty for the Treatment of Localized Eosinophilic Otitis Media in the Tympanic Cavity: A Report of Two Cases

**DOI:** 10.7759/cureus.90405

**Published:** 2025-08-18

**Authors:** Kokushi Wake, Takenori Miyashita, Satoshi Takahashi, Hiroshi Hoshikawa

**Affiliations:** 1 Department of Otolaryngology, Faculty of Medicine, Kagawa University, Miki, JPN; 2 Department of Otolaryngology, Takamatsu Red Cross Hospital, Takamatsu, JPN

**Keywords:** biologic, eardrum reconstruction, endoscopic ear surgery, eosinophilic otitis media, tympanoplasty

## Abstract

Eosinophilic otitis media (EOM) is a form of refractory otitis media characterized by eosinophil-dominant, highly viscous middle ear effusion, for which tympanoplasty is generally contraindicated. In patients with well-controlled otorrhea who do not require transtympanic therapy, tympanic membrane closure may significantly improve clinical severity scores and reduce the risk of infection.

We report two cases of EOM localized to the mesotympanic and hypotympanic cavities that were successfully treated with transcanal endoscopic tympanoplasty without the use of biologics. In both cases, the procedure improved hearing and severity scores, resolved otorrhea, suppressed infection, and alleviated mucosal irritation in the middle ear. Reconstruction of the tympanic membrane using auricular cartilage helped prevent adhesion to mucosal defect regions in the middle ear.

These cases suggest that transcanal endoscopic tympanoplasty may be effective in the absence of complications such as chronic eosinophilic rhinosinusitis in patients with well-controlled otitis media and localized mucosal lesions. This report demonstrates the potential applicability of tympanoplasty in selected patients with EOM, despite traditional contraindications. Furthermore, it highlights the effectiveness of combining three antiallergic agents (ibudilast, montelukast, and cetirizine) in controlling postoperative eosinophilic inflammation.

## Introduction

Eosinophilic otitis media (EOM), characterized by an eosinophil-dominant, highly viscous middle ear effusion, is a form of refractory otitis media in which tympanoplasty is contraindicated [1-4]. Although rare, surgery for EOM can occasionally result in worsening bone conduction hearing or, in a few cases, deafness. The diagnostic criteria proposed by Iino et al. include otitis media with eosinophil-predominant effusion or granulation, along with minor criteria of highly viscous middle ear effusion, resistance to treatment, bronchial asthma, and nasal polyposis [2].

EOM is considered a type 2 inflammatory disease of the middle ear [5]. Triamcinolone acetonide instillation into the tympanic cavity has been reported as an effective treatment for patients with EOM [5]. Moreover, a combination of various antiallergic drugs has been employed to reduce the frequency of steroid instillations in patients with EOM [6,7]. The hearing loss associated with EOM is initially conductive; however, the bone conduction threshold gradually deteriorates as the disease progresses, often resulting in mixed hearing loss. Risk factors for elevated bone conduction thresholds include prolonged disease duration, tympanoplasty, advanced middle ear mucosal lesions, and bacterial infection [5].

Surgical treatment of EOM using tympanoplasty with mastoidectomy is generally contraindicated due to the potential risk of elevated bone conduction thresholds and exacerbation of inflammation [2]. In patients with long-term control of otorrhea, myringoplasty has been reported to significantly improve clinical severity scores [8,9]. Tympanoplasty has been suggested to be useful in patients with long-term otorrhea control using biologics, as the infection risk can be reduced by closing the tympanic membrane perforation [5]. Transcanal endoscopic tympanoplasty requires minimal bone resection and is considered one of the most suitable surgical options for EOM localized in the tympanic cavity.

Here, we present two representative cases of EOM localized in the tympanic cavity, with favorable outcomes following transcanal endoscopic tympanoplasty without the use of biologics.

## Case presentation

Case 1

Background

**Figure 1:**
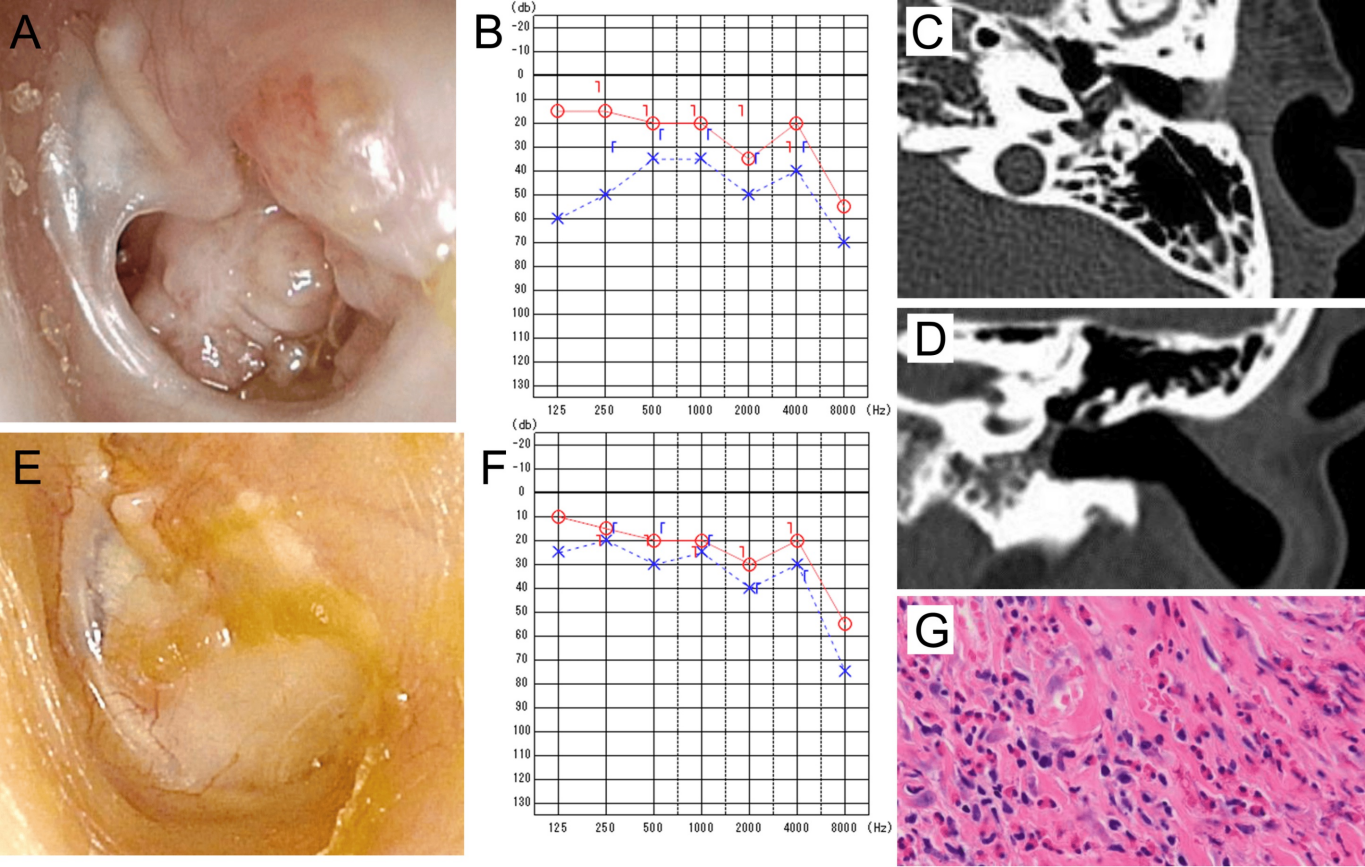
Pre- and postoperative clinical findings of case 1 A: Preoperatively, a large perforation of the left tympanic membrane was observed, and middle ear granulation tissue protruded beyond the edge of the perforation into the external auditory canal. B: Preoperative pure tone audiometry revealed conductive hearing loss in the left ear. C and D: Axial (C) and coronal (D) preoperative temporal bone CT images. A soft tissue shadow consistent with granulation tissue was observed in the mesotympanic and hypotympanic cavities. E: Postoperative view of the tympanic membrane. The perforation was successfully closed, and otorrhea had resolved. F: Postoperative pure tone audiometry revealed an improvement in conductive hearing loss in the left ear. G: Histopathological findings of granulation tissue harvested during surgery showing numerous eosinophils (~150/HPF) as the predominant inflammatory cells. CT: computed tomography, HPF: high-power field

A 50-year-old woman presented with a history of intermittent otorrhea in the left ear, which had been diagnosed as chronic otitis media during childhood. The patient had no history of bronchial asthma and visited her previous physician due to increased otorrhea. She was referred to our hospital for further surgical evaluation. Initial examination revealed a large left tympanic membrane perforation, granulation tissue protruding from the middle ear, and highly viscous yellow otorrhea (Figure 1A).

Cytological analysis of the otorrhea identified eosinophils, confirming the diagnosis of EOM. Audiometry revealed conductive hearing loss of 40 dB in the left ear (bone conduction: 28.3 dB) (Figure 1B). Computed tomography (CT) demonstrated soft tissue shadows in the left mesotympanic and hypotympanic cavities (Figure 1C, 1D), with no abnormalities detected in the eustachian tube, epitympanic cavity, or mastoid air cells.

Surgery

**Figure 2:**
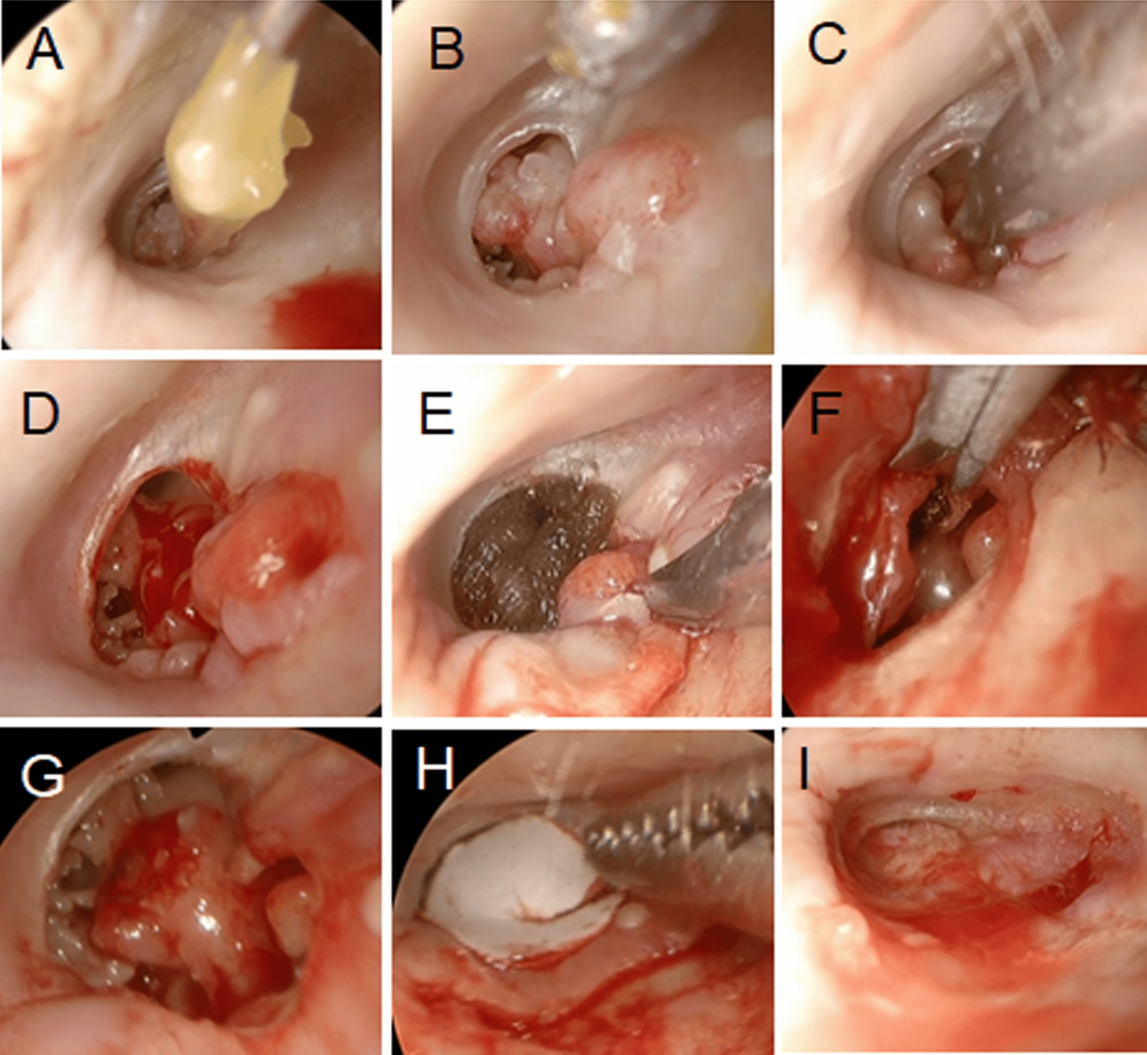
Surgery of case 1 A: A highly viscous, yellow otorrhea was removed from the inferior tympanic cavity. B: Multiple granulation tissues were observed protruding from the promontory and periauricular area into the external auditory canal. C: The granulation tissue on the promontory was excised without exposure of the bony wall of the middle ear. D and E: Scar tissue surrounding the tympanic membrane perforation was excised. F and G: Granulation tissue on both the lateral and medial surfaces of the tympanic membrane was excised. H: The shape and size of the tympanic membrane perforation were measured using a template. I: The membrane was reconstructed using the underlay technique with auricular cartilage, shaped to fit the template.

The base of the granulation tissue protruding from the middle ear was identified and excised without exposing the bony surface (Figure 2).

Scar tissue surrounding the tympanic membrane perforation was excised. The posterior wall of the external auditory canal was incised, and the tympanomeatal flap was elevated. Granulation tissue on the tympanic membrane was dissected and excised to avoid bony exposure and preserve the tympanic nerve. Granules surrounding the ossicles were also removed. Tympanic membrane reconstruction was performed using the underlay technique with auricular cartilage, shaped with gradually thinning margins and fixed in place with fibrin glue (Figure 2).

Pre- and Postoperative Course

Histopathological analysis of the middle ear granulation tissue by pathologists demonstrated eosinophil-predominant inflammation, confirming the diagnosis of EOM in accordance with the major criteria of established diagnostic guidelines (Figure 1G). The EOM severity score improved from 3 to 0 (Table 1).

**Table 1:** Severity scores of EOM The EOM severity scoring system, originally developed and published by Iino et al. [8-10], is a publicly available tool that assesses disease activity using five clinical parameters, each scored from 0 to 2. Categories A and B assess the extent of local inflammation in the middle ear, while Categories C, D, and E reflect the intensity of treatment interventions. The preoperative severity scores for cases 1 and 2 were 3 and 4, respectively, indicating that eosinophilic inflammation was confined to the middle ear in both patients. Following transcanal endoscopic tympanoplasty, the severity scores improved to 0 in both cases, suggesting complete clinical resolution. EOM: eosinophilic otitis media, MEE: middle ear effusion Partially modified from Iino et al. [10]

Category	Case 1	Case 2
A: The mucosal condition (0: nearly normal or slightly edematous, 1: edematous or slightly thickened, 2: highly thickened or granulated to an extent beyond the position of a normal eardrum)	1	2
B: The quantity of MEE/otorrhea (0: no MEE, 1: MEE with intratympanic aeration in a case without eardrum perforation or otorrhea limited to the mesotympanum in a case with perforation, 2: mesotympanum totally filled with MEE in a case without perforation or otorrhea coming out from the mesotympanum to the external auditory canal in a case with perforation)	2	2
C: The frequency of intratympanic administration of corticosteroid (0: none, 1: once in the previous 3 months, 2: two or more times in the previous 3 months)	0	0
D: The frequencies of systemic administration of corticosteroids (0: none, 1: 7 days or less in the previous 3 months, 2: more than 7 days in the previous 3 months)	0	0
E: The frequencies of systemic antibiotics (0: none, 1: 7 days or less in the previous 3 months, 2: more than 7 days in the previous 3 months)	0	0

Ibudilast, cetirizine hydrochloride, and montelukast sodium were administered postoperatively. The patient's hearing improved, with no recurrence of otorrhea over a three-year follow-up period (Figure 1E, 1F).

Case 2

Background

**Figure 3:**
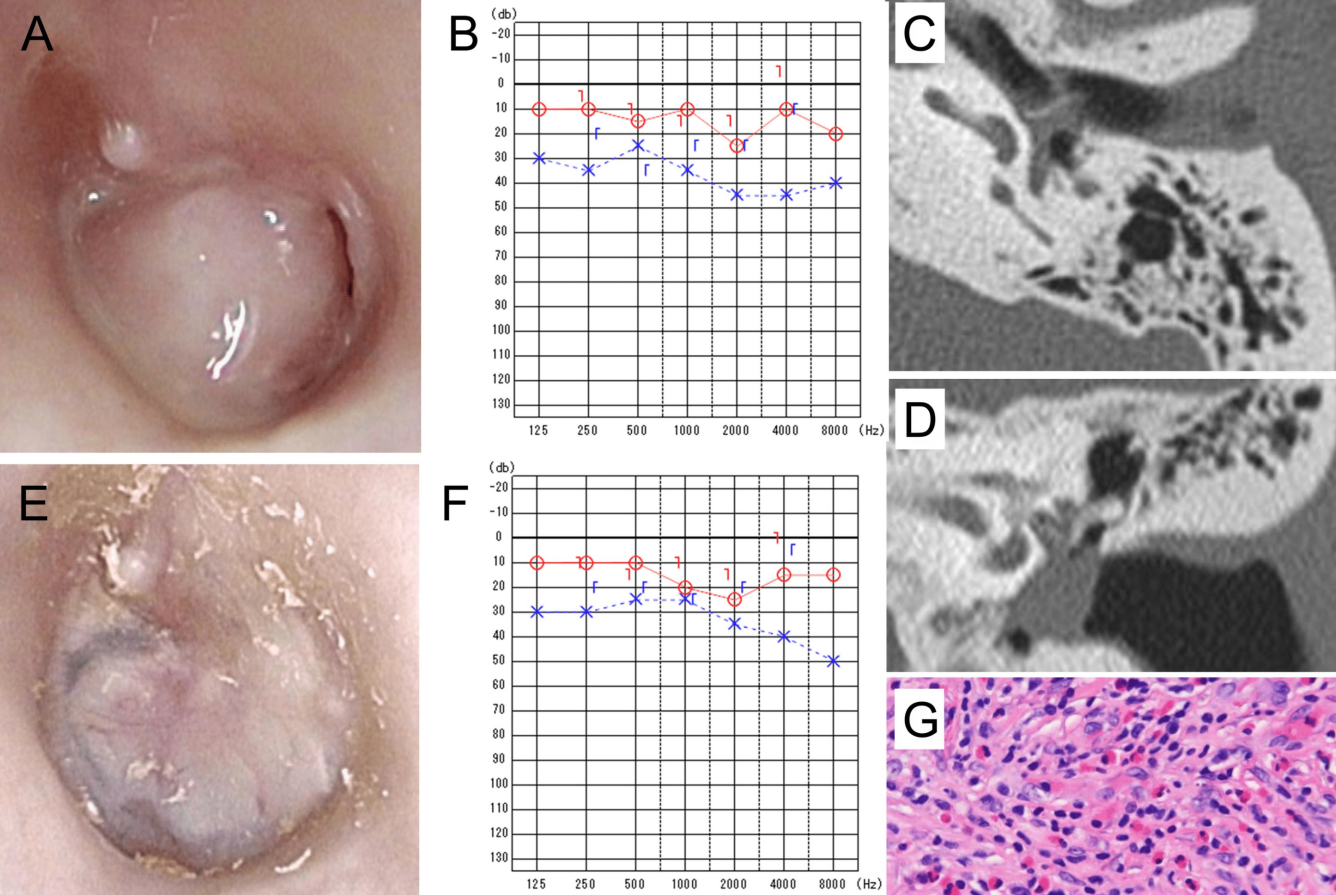
Pre- and postoperative clinical findings of case 2 A: Preoperatively, a large perforation of the left tympanic membrane was observed, and prominent granulation tissue protruded beyond the edge of the perforation into the external auditory canal. B: Preoperative pure tone audiometry revealed hearing loss in the left ear. C and D: Axial (C) and coronal (D) preoperative temporal bone CT images. Soft tissue shadows consistent with granulation tissue were seen in the middle ear and subtympanic cavities. E: Postoperatively, the tympanic membrane perforation was closed, tympanic membrane retraction was prevented, and no otorrhea was observed. F: Postoperative pure tone audiometry demonstrated improved hearing in the left ear. G: Histopathological findings of granulation tissue harvested during surgery showing numerous eosinophils (~100/HPF) as the predominant inflammatory cells. CT: computed tomography, HPF: high-power field

A 40-year-old woman with a history of type 2 diabetes and hypertension presented with otorrhea in the left ear, which had been diagnosed as a perforated tympanic membrane during childhood but remained untreated. She visited a local clinic with a chief complaint of otorrhea and was referred to our hospital after granulation tissue was found filling the left tympanic cavity. On initial examination, granulation tissue was seen protruding from the left middle ear beyond the margin of the perforated tympanic membrane into the external auditory canal (Figure 3A).

Bacteriological analysis of the otorrhea identified *Staphylococcus aureus*. Audiometry showed conductive hearing loss of 43.4 dB in the left ear (bone conduction: 26.7 dB) (Figure 3B). The patient was diagnosed with chronic suppurative otitis media due to bacterial infection. Following successful control of otorrhea, transcanal endoscopic tympanoplasty was planned to prevent recurrence. CT imaging revealed soft tissue shadows in the mesotympanic and hypotympanic cavities (Figure 3C, 3D), with some involvement around the ossicles, but no abnormalities in the mastoid cavity or air cells.

Surgery

Scar tissue surrounding the tympanic membrane perforation was excised. The posterior wall of the external auditory canal was incised, and the tympanomeatal flap was elevated. The tympanic nerve was preserved to maintain access to the middle ear cavity. The base of the middle ear polyp was located at the promontory and was excised without exposing the bony surface. Tympanic membrane reconstruction was performed using the underlay technique with two slices of auricular cartilage (1/2 inch thick), arranged in a palisade fashion and fixed with fibrin glue. The tympanomeatal flap was repositioned and sealed with fibrin glue.

Postoperative Course

Histopathological analysis of the middle ear granulation tissue by pathologists revealed eosinophil-predominant inflammation, confirming the diagnosis of EOM in accordance with the major criteria of established diagnostic guidelines (Figure 3G). At one-year follow-up, the patient's hearing had improved, and no recurrence of otorrhea was observed (Figure 3E, 3F). The EOM severity score improved from 4 to 0 (Table 1).

## Discussion

Transcanal endoscopic tympanoplasty is a minimally invasive surgical approach to resect granulation tissue without exposing the bony surface of the middle ear in cases of EOM localized to the tympanic cavity. In both cases, transcanal endoscopic tympanoplasty led to hearing improvement, cessation of otorrhea, infection control, and reduction in mucosal irritation, resulting in favorable outcomes. These findings suggest that this technique may be effective in patients with well-controlled otitis media, middle ear mucosal lesions limited to the mesotympanic and hypotympanic regions, and absence of complications such as chronic eosinophilic rhinosinusitis.

Previous studies have shown that surgery on the EOM, especially when it involves exposing bone, such as in mastoidectomy, can sometimes lead to worse bone conduction hearing or, in rare cases, to severe hearing loss [5]. Proposed mechanisms include exacerbation of inner ear inflammation due to the spread of eosinophil-rich effusion through the round window membrane and iatrogenic labyrinthitis. Given this inherent risk, it is crucial to avoid extensive drilling or bony exposure whenever possible.

Closure of the tympanic membrane perforation in eosinophilic otitis media (EOM) offers several advantages, including a reduced risk of trans-eustachian infections and the potential for hearing improvement [8,9]. However, such closure may also hinder the delivery of intratympanic corticosteroid therapy by eliminating a direct route of administration, thereby complicating local treatment strategies [8,9]. Proposed indications for myringoplasty in EOM include minimal middle ear effusion or otorrhea with stable hearing levels in the absence of intratympanic corticosteroid use, no recent systemic administration of corticosteroids or antibiotics, and the absence of pathologic mucosal changes or granulation tissue extending into the external auditory canal [8,9]. Conversely, myringoplasty is contraindicated in cases involving persistent otorrhea, extensive mucosal pathology, or total tympanic membrane perforation [8,9].

In the present two cases, criteria for minimal effusion and lack of systemic treatment were satisfied; however, the third condition (absence of extensive mucosal pathology) was not met, as both patients exhibited severe middle ear mucosal involvement and total perforation, which would typically preclude surgical intervention. Nonetheless, based on a comprehensive analysis of these cases and prior reports [5,8,9], we propose that transcanal endoscopic tympanoplasty may still be a viable treatment option when the following conditions are met: limited middle ear effusion or otorrhea with stable hearing without intratympanic corticosteroid administration, no systemic use of corticosteroids or antibiotics, mucosal lesions confined to the mesotympanic and hypotympanic regions, and well-aerated mastoid and mastoid air cells on computed tomography imaging.

A diagnosis of EOM is confirmed when the major criterion and at least two minor criteria are met [2]. In case 1, the diagnosis was established preoperatively, allowing for the intraoperative preparation of steroids and antihistamines. Case 2 presented with symptoms suggestive of chronic otitis media, including viscous otorrhea and infected granulation tissue. Postoperative histopathology revealed eosinophil-dominant inflammation, thereby confirming EOM. In both cases, antibiotics and local steroid administration failed to control the granulation. Surgical excision of the granulation tissue and closure of the tympanic membrane perforation effectively reduced mucosal irritation and achieved inflammatory control. Eosinophilic inflammation was considered confined to the middle ear. These cases represent localized, atypical forms of EOM without systemic eosinophilic comorbidities and are pathologically characterized by eosinophil-predominant inflammation.

Following surgery, case 2 did not require additional treatment. In contrast, case 1 exhibited a moist tympanic membrane postoperatively and was treated with ibudilast, montelukast, and cetirizine. This led to drying of the tympanic membrane and effective disease control. Antiallergic agents that suppress eosinophilic inflammation include cysteinyl leukotriene receptor antagonists (montelukast), phosphodiesterase 4 inhibitors (ibudilast), anti-H1 receptor antagonists (cetirizine), and dual antagonists of thromboxane A2 and prostaglandin D2 receptors (ramatroban) [5-7]. Matsubara et al. reported that combining two or three antiallergic drugs effectively reduced the frequency of intratympanic steroid injections in patients with EOM [7]. In case 1, eosinophilic inflammation was well controlled using a combination of three oral antiallergic agents, consistent with previous findings [7].

Iino et al. developed a severity scoring system for EOM that evaluates five clinical parameters on a scale from 0 to 2 (Table 1) [8-10]. This scoring system has been shown to be useful in evaluating the efficacy of EOM treatment [8-13]. In the present cases, severity scores improved from 3 to 0 in case 1 and from 4 to 0 in case 2. Both patients exhibited localized eosinophilic inflammation without systemic complications such as asthma or eosinophilic sinusitis. Elevated scores were observed in the categories of middle ear effusion/otorrhea volume and mucosal consistency, while scores for the remaining three categories were zero. The EOM severity score is considered effective in assessing the localization and severity of eosinophilic inflammation and evaluating surgical outcomes. According to this score, transcanal endoscopic tympanoplasty may be a viable treatment option when the scores for the items frequency of intratympanic corticosteroid administration, systemic corticosteroid use, and antibiotic use in the preceding three months are all zero.

## Conclusions

This study presents two cases of eosinophilic otitis media (EOM) in which transcanal endoscopic tympanoplasty was successfully performed for localized eosinophilic inflammation confined to the middle ear. Both patients exhibited improvement in hearing and complete resolution of otorrhea, with postoperative EOM severity scores reduced to 0. These findings demonstrate that when specific clinical conditions are met, such as minimal prior corticosteroid use, absence of systemic complications, and localized disease, transcanal endoscopic tympanoplasty is a viable and effective treatment option. The EOM severity scoring system was instrumental in identifying appropriate surgical indications and quantifying treatment outcomes, thereby supporting its continued use in clinical decision-making for EOM.
